# Reviewed article: Carvalho, SMO; Andrade, JX; Nascimento, FF; Rodrigues, MTP; Mascarenhas, MDM. Temporal trends in acquired syphilis detection, hospitalizations, and mortality: a time series analysis, Brazil, 2011-2023. Epidemol Serv Saude. 2026:35;e20251388

**DOI:** 10.1590/S2237-96222026v35e20251388.c

**Published:** 2026-11-02

**Authors:** Mariana Del Grossi Moura

**Affiliations:** 1 Universidade de Sorocaba, Sorocaba, SP, Brazil Universidade de Sorocaba Sorocaba SP Brazil

## First round comments

Agradecemos a submissão do manuscrito RESS-2026-1388 intitulado “Tendência temporal da detecção, internação hospitalar e mortalidade da sífilis adquirida, Brasil, 2011-2023: estudo de série temporal” à Revista Epidemiologia e Serviços de Saúde: revista do SUS (RESS).

Após a revisão do seu artigo, identificamos a necessidade de uma revisão substancial para que o manuscrito esteja de acordo com os padrões editoriais da revista e para viabilizar sua publicação.

Além dos pontos detalhados pelos revisores, há necessidade dos seguintes ajustes:

1. Título: o título deve informar o tema principal, delineamento, local e ano(s) da pesquisa (nessa ordem). Sugestão: Tendência temporal da detecção, internações hospitalares e mortalidade da sífilis adquirida: análise de série temporal, Brasil, 2011-2023;
2. Delineamento do estudo (Resumo e texto completo): substituir para “Análise de série temporal, com dados secundários [...]”
3. Método (resumo): dados secundários disponíveis na página eletrônica do Departamento de Informática do Sistema Único de Saúde parece genérico. Especificar os sistemas de informação (ex.: SINAN, SIH/SUS, SIM); preferir “Foram calculadas”, “Foi utilizado” ao invés de “Calcularam-se”, “Utilizou-se”;
4. Resultados (resumo): padronizar “por 100 mil habitantes”; “taxa média de internação hospitalar” ao invés de “taxa média de internação”; “taxa de detecção” ao invés de “detecção”; “taxa de mortalidade” ao invés “mortalidade”; “no Brasil” repetido em todas as frases, manter na primeira menção e suprimir nas subsequentes quando não houver ambiguidade; revisar os métodos, mantendo apenas as variáveis efetivamente apresentadas nos resultados ou incluir, nos resultados, ao menos os principais achados referentes às estratificações por sexo e faixa etária, caso essas análises sejam relevantes para o objetivo do estudo;
5. Resumo (conclusão): Manter a conclusão centrada nos achados observados, evitando introduzir recomendações não derivadas diretamente da análise apresentada no resumo;
6. Palavras-chave: substituir o termo “Incidência”, uma vez que o estudo utiliza taxas de detecção, e não estimativas de incidência propriamente ditas; o delineamento do estudo deve ser mantido como o último descritor;
7. Introdução: iniciar com “A sífilis é uma condição prevenível e tratável, mas permanece um desafio para a saúde pública...”; apresentar o objetivo de forma direta por exemplo: Diante desse cenário, o estudo teve por objetivo analisar a tendência temporal da detecção de sífilis adquirida, das internações hospitalares por sífilis adquirida e da mortalidade por sífilis adquirida no Brasil, no período de 2011 a 2023.”
8. Métodos (texto principal): Recomenda-se aderir ao checklist STROBE, organizando o relato conforme os itens: delineamento, contexto, participantes, variáveis, fontes de dados/mensuração, viés, tamanho do estudo e métodos estatísticos;
9. Método (delineamento): substituir por “análise de série temporal”;
10. Método: incluir descrição de limitações inerentes ao uso de dados secundários (ex.: subnotificação, inconsistências nos registros); incluir “Tamanho do estudo” explicitar que o estudo incluiu todos os registros disponíveis no período analisado;
11. Método (Fontes de dados e mensuração): o SINAN registra casos notificados, e não necessariamente todos os casos diagnosticados. Ajustar redação; substituir “infecção” por “sífilis adquirida”
12. Método (métodos estatísticos): p-valor descrito duas vezes “O nível de significância estatística foi adotado quando p-valor<0,050.” e “O p-valor, obtido para o coeficiente de regressão (β), foi utilizado para determinar a significância estatística da tendência observada, adotando-se o nível de significância quando o p-valor<0,050.”;
13. Resultados: padronizar unidade “por 100 mil habitantes”; padronizar “taxa média de internação hospitalar”; não inclua interpretações como “crescimento expressivo”, “particularmente elevados”, “queda abrupta”... substituir por descrição direta dos dados; “tendência crescente” repetida em excesso; em VPA 9,19; IC95%: 6,21; 12,26 remover “:” deixando apenas o espaço; checar consistência com resumo “13.036 internações” vs “13.336 internações”; padronizar “detecção de sífilis” vs “taxa de detecção” e “taxa de internação” vs “taxa de internação hospitalar”; verificar trecho “grupo pessoas a com 60 ou mais anos de idade” com ajuste para “grupo de pessoas com 60 anos ou mais”; remover interpretação causal referência à pandemia, manter “redução observada no ano de 2020”;
14. Resultados tabela 1: verificar trechos descritivos que resumem os resultados (ex.: “incremento superior a 50%”), assegurando que esses valores estejam de fato representados nas tabelas (VPA ≥50%);
15. Tabela 1, 2 e 3: simplificar “janeiro/2011 a dezembro/2023” para 2011-2023; padronizar “Faixa etária (anos)”; padronizar o alinhamento dos dados na tabela, mantendo os valores numéricos alinhados à direita e os textos, como nomes de variáveis e categorias, alinhados à esquerda; substituir “N” por “n” nos cabeçalhos;
16. Tabela 2: total 13.036, porém Feminino + masculino: 6.280 + 6.737 = 13.017 com diferença de 19 registros (precisa de nota de rodapé); Região Norte também com inconsistência numérica (subgrupos > total); uso de “<0,000” é incorreto, usar “<0,001”; incluir nota de rodapé;
17. Tabela 3: incluir nota de rodapé para inconsistência numérica (sexo e faixa etária);
18. Figura 1: o uso de “Evolução” é genérico e pode ser suprimido; simplificar “janeiro/2011 a dezembro/2023” para 2011-2023; corrigir a identificação dos painéis, substituindo a repetição incorreta de “A” por “C” no gráfico de mortalidade; melhorar a legibilidade da figura, com aumento do tamanho das fontes e ajuste da legenda para facilitar a identificação dos grupos; revisar o espaçamento e a organização dos painéis, considerando a alta densidade de informações na figura; para figuras compostas (mosaicos): identifique cada parte com letras maiúsculas e descreva-as na legenda, em texto completo de sentido (ex.: “Consumo de medicamentos em homens (A) e mulheres (B)”). Preferencialmente empregue a mesma escala nas figuras que compõem o mosaico; minimize o número de elementos dentro do campo de dados e certifique-se de que todos estejam claramente identificados;
19. Figura 1: Em caso de aceite, as figuras devem ser encaminhadas em arquivos separados para diagramação. Gráficos, mapas e demais figuras devem ser enviados no formato PDF, SVG e EPS, exportadas em um dos formatos diretamente do software utilizado na sua criação. Gráficos criados em Excel podem ser enviados no formato XLSX. Em caso de fotos, a resolução mínima é de 300 dpi, no formato JPEG.
20. Discussão: esta longa, sugestão de reestruturação - O primeiro parágrafo da discussão deve resumir os achados, interpretando-os e sem repetir números nem citar ilustrações ou referências: não informar o que fez, interpretar o que encontrou. Apresentar as limitações do estudo, levando em consideração fontes potenciais de viés ou imprecisão no segundo parágrafo. Após esclarecer as limitações da pesquisa, o texto da discussão deve ser guiado pelos seus achados. Interpretar cada resultado relevante e discuti-lo à luz da literatura. Esgotar o tema abordado e evitar retomá-lo adiante no texto de modo circular e redundante. Evitar parágrafos sem conexão com a literatura ou que somente a revisam, sem discussão com os resultados. Assegurar que a discussão faz a ponte entre os achados e pesquisas compatíveis - com delineamento, amostragem e demais aspectos metodológicos comparáveis ao que foi realizado na pesquisa -, ou evidenciar as discrepâncias; caracterizar o estudo citado (delineamento, pessoa, tempo e lugar) de modo a esclarecer semelhanças e diferenças pertinentes a tal confrontação. O último parágrafo (curto e conciso) deve apresentar a conclusão da pesquisa com base do que se pode afirmar a partir dos resultados;
21. Discussão: reduzir o uso de termos interpretativos e evitar extrapolações não diretamente sustentadas pelos resultados, especialmente explicações causais ou comportamentais;
22. Referências: a RESS recomenda incluir DOI sempre que disponível; revisar seguindo consistentemente as regras do estilo Vancouver, disponível em: http://www.nlm.nih.gov/bsd/uniform_requirements.html.

Solicitamos que todos os comentários da revisão por pares sejam respondidos pontualmente por meio de uma carta resposta detalhada, explicando as modificações realizadas ou justificando, quando necessário, a manutenção do texto original. A nova versão do manuscrito será reconsiderada após essa reformulação e poderá passar por nova rodada de avaliação. Destacamos que esta decisão não garante a aceitação final do manuscrito.

Recommendation: Major revision 

## Second round comments

O manuscrito apresentou avanço substancial após a revisão, especialmente quanto à organização metodológica, padronização do delineamento, detalhamento das fontes de dados e clareza da redação científica. As principais recomendações dos revisores foram incorporadas de forma satisfatória.

Antes da decisão editorial final, recomenda-se apenas uma revisão adicional voltada principalmente a ajustes editoriais e de padronização, com atenção aos seguintes pontos:

- revisar o objetivo da introdução para evitar repetição excessiva da expressão “sífilis adquirida”;
- simplificar trechos excessivamente interpretativos, priorizando linguagem mais descritiva e diretamente sustentada pelos resultados;
- revisar a discussão para reduzir redundâncias e tornar os parágrafos mais objetivos, especialmente nas descrições regionais;
- revisar cuidadosamente as tabelas para correção de inconsistências numéricas, erros de digitação, alinhamento e uniformidade de formatação;
- revisar pequenas inconsistências gramaticais e de fluidez textual;
- padronizar integralmente expressões como “taxa de detecção”, “taxa de internação hospitalar” e “taxa de mortalidade” ao longo de todo o manuscrito;

De forma geral, o manuscrito encontra-se metodologicamente consistente, com adequada articulação entre objetivo, métodos, resultados e discussão, apresentando contribuição relevante para a temática proposta.

A nova versão do manuscrito será reconsiderada após essa reformulação.

Recommendation: Minor revision 

## Third round comments

Agradeço o envio da versão revisada do manuscrito RESS-2025-1388.R2, intitulado “Tendência temporal da detecção, internações hospitalares e mortalidade da sífilis adquirida: análise de série temporal, Brasil, 2011-2023”.

O manuscrito apresentou avanços importantes, mas antes da decisão editorial final, solicito atenção aos seguintes ajustes:

A repetição da expressão “sífilis adquirida” foi corrigida no resumo; contudo, o objetivo apresentado na introdução ainda apresenta repetição excessiva desse termo. Solicita-se harmonizar a redação entre as diferentes seções do manuscrito.

A discussão está organizada e compreensível, porém permanece extensa em alguns trechos. Recomenda-se reduzir repetições nas descrições regionais, especialmente em relação às explicações para o aumento dos indicadores e à necessidade de fortalecimento das ações de controle, tornando os parágrafos mais objetivos. Recomenda-se também limitar as interpretações ao que foi efetivamente avaliado no estudo, evitando atribuir os achados a fatores não investigados diretamente.

Padronizar o número de casas decimais nos métodos, resultado e ilustrações: percentual 1 casa decimal, medida de associação: 2 casas decimais, p-valor: 3 casas decimais. Modelo: IC95% 1,14; 2,23;

Solicita-se também o envio de carta-resposta indicando, de forma objetiva, as alterações realizadas no manuscrito ou a justificativa para sua não realização.

Recommendation: Minor revision 

